# Metagenomic Analysis of Microbial Diversity in Cotton Rhizosphere Soil in Alwar, India

**DOI:** 10.1128/MRA.00987-20

**Published:** 2020-12-03

**Authors:** Rajeshwar Pratap Singh, Atul Kumar Johri, Meenakshi Dua

**Affiliations:** a Microbial Ecology Laboratory, School of Environmental Sciences, Jawaharlal Nehru University, New Delhi, India; b School of Life Sciences, Jawaharlal Nehru University, New Delhi, India; Georgia Institute of Technology

## Abstract

Cotton is an important cash crop for both the Indian economy and rural livelihoods. In the present study, metagenomic analysis is used to characterize microbial diversity in cotton rhizosphere soil from the Alwar district, located in the semiarid northeast region of the state of Rajasthan in India.

## ANNOUNCEMENT

The rhizosphere microbiome contains many beneficial microbes that are closely associated with plant growth and productivity. Therefore, study of this microbiome is crucial for improving plant productivity in a sustainable manner. Next-generation sequencing technologies using whole genomes from environmental samples provide useful insights in microbiome studies (1). Cotton (*Gossypium* spp.) is an important cash crop for which India cultivates the greatest acreage in the world. It makes up 65% of the raw material requirements for the Indian textile industry (2). Environmental conditions, especially temperature and water availability, have significant roles in cotton production by influencing phenological crop events, i.e., squaring, flowering, ball formation, and ball opening (3). It has been demonstrated that cotton productivity is linked to the diverse microbial community hosted in its rhizosphere (4).

Cotton rhizosphere soils were collected 0 to 5 cm around the cotton root at a depth of 0 to 30 cm with a field soil corer in November 2017. Wet soil samples were collected from four random points in a field in the semiarid region of Navgavan village in the Alwar district of Rajasthan, India (27.5259N, 76.1548E). All of the samples were evenly mixed and sieved through 0.2-mm mesh to prepare a composite sample. Genomic DNA was extracted using the NucleoSpin soil kit (TaKaRa Bio, USA). Paired-end (PE) sequencing libraries were prepared using the Illumina TruSeq Nano DNA library preparation kit. The size-selected product was PCR amplified, and the enriched libraries were analyzed on a 4200 TapeStation system (Agilent Technologies). Illumina PE libraries with a mean fragment size of 471 bp were sequenced on a NextSeq 500 system using 2 × 150-bp chemistry. The PE raw reads were processed using Trimmomatic v0.38 (5) to obtain 15,229,497 high-quality reads. The high-quality reads were assembled into scaffolds using CLC Genomics Workbench v9.5.2 6 (Qiagen). Genes were predicted from the assembled scaffolds using Prodigal v2.6.3 (6). Taxonomic analysis of predicted genes was carried out using Kaiju v1.6.2 (7). Functional analysis to provide gene annotations through the Cluster of Orthologous Groups (COG) and KEGG databases was carried out using COGNIZER v0.9b (8). Default parameters were used for all software except where otherwise noted.

Of the 185,231 assembled scaffolds (assembly size, 90,525,445 bp; average size, 489 bp; *N*_50_, 458 bp), 229,401 genes were predicted, with an average gene length of 351 bp. Approximately 25% of the reads could not be identified taxonomically. Among the 75% identified reads, *Bacteria* (70%) dominated the microbial diversity, while *Eukaryota* (2%) and *Archaea* (3%) formed very small fractions. The most abundant *Bacteria* phylum was *Proteobacteria* (32.95%), represented strongly by Acinetobacter (5.36%). The other phyla, in order of abundance, were *Acidobacteria* (17.14%), *Actinobacteria* (14.38%), *Bacteroidetes* (6.28%), *Gemmatimonadetes* (6.27%), and *Chloroflexi* (3.32%). *Nitrososphaera* (3.39%) was the most abundant *Archaea* genus found present. Functional classification using the COG database distributed genes into general function (15%), amino acid transport and metabolism (11%), energy production and conversion (7%), carbohydrate transport and metabolism (6%), and transcription (6%), among others. Ontology analysis based on the KEGG database distributed genes into metabolism (38%), environmental information processing (11%), cellular processes (3%), and genetic information processing (6%). The genes and pathways identified are only predictions. For cotton rhizosphere, a similar microbial profile was reported earlier (9).

### Data availability.

This whole-genome shotgun project has been deposited in GenBank under the accession number JADBHV000000000. The version described in this paper is version JADBHV010000000. The raw metagenome sequence has been deposited in the NCBI Sequence Read Archive (SRA) under the accession number SRR12376372. The BioSample accession number is SAMN15693165.
